# Complete Genome Sequence of *Exiguobacterium* sp. Strain N4-1P, a Psychrophilic Bioemulsifier Producer Isolated from a Cold Marine Environment in North Atlantic Canada

**DOI:** 10.1128/genomeA.01248-17

**Published:** 2017-11-02

**Authors:** Qinhong Cai, Xudong Ye, Bing Chen, Baiyu Zhang

**Affiliations:** The Northern Region Persistent Organic Pollution Control (NRPOP) Laboratory, Faculty of Engineering and Applied Science, Memorial University of Newfoundland, St. John’s, Newfoundland, Canada

## Abstract

Here, we present the complete genome sequence of *Exiguobacterium* sp. strain N4-1P, a psychrophilic bacterium that produces bioemulsifier, isolated for the first time from petroleum hydrocarbon-contaminated sediment samples from shoreline Newfoundland, Canada. Many strains of the genus *Exiguobacterium* are extremophiles and have properties of biotechnological interest.

## GENOME ANNOUNCEMENT

*Exiguobacterium* sp. strain N4-1P is a psychrophilic facultative anaerobic bacterium isolated from shoreline sediment samples contaminated by petroleum hydrocarbon in Newfoundland, Canada, by using *n*-hexadecane or diesel as the sole carbon source (1). This was the first *Exiguobacterium* strain found to be capable of using hydrocarbon as the sole carbon source and producing surface active agents to stabilize oil-water emulsions (1, 2). According to 16S DNA phylogenetic analysis, *Exiguobacterium oxidotolerans* sp. strain sN4-1P is closely related to *E. oxidotolerans* strain T-2-2^T^, *Exiguobacterium antarcticum* B7^T^, and *Exiguobacterium sibiricum* 255-15^T^ with 99.47%, 98.70%, and 98.63% similarities, respectively (2).

The genus *Exiguobacterium* consists of Gram-positive facultative anaerobes with low G+C content (3). *Exiguobacterium* species have been isolated from diverse habitats over a wide temperature range (−12 to 55°C), such as glacial ice, hot springs, Siberian permafrost, and tropical soils (4). *Exiguobacterium* strains possess interesting features, such as temperature acclimation proteins and enzymes, which have potential applications in the food and pharmaceutical industries and in environmental remediation (3, 4). However, genomic investigations of *Exiguobacterium* spp. have been limited (3).

Whole-genome shotgun sequencing of *Exiguobacterium* sp. strain N4-1P was performed at the Donnelly Sequencing Center at the University of Toronto (Toronto, Canada) using an Illumina MiSeq 2500 with a 300-cycle MiSeq kit V2. This generated a total of 2,260,831 filtered paired-end reads, providing 233-fold coverage of the genome. Quality control was conducted through FastQC (http://www.bioinformatics.babraham.ac.uk/projects/fastqc). To assemble the data, SPAdes genome assembler was used (5). plasmidSPAdes was used to assemble plasmids from whole-genome sequencing data (6). Subsequently, the assembled contigs were reordered by Mauve multiple-genome alignment using *Exiguobacterium antarcticum* B7 as the reference genome (7). Gene annotation was performed by using the NCBI Prokaryotic Genome Annotation Pipeline (http://www.ncbi.nlm.nih.gov/genome/annotation_prok/), and tRNA and rRNA sequences were identified using tRNAscan-SE and RNAmmer, respectively (8, 9).

Finally, a chromosome type replicon (3,032,448 bp with G+C content of 46.8%) made of 54 contigs/scaffolds was obtained for *Exiguobacterium* sp. strain N4-1P, which harbors 3,112 coding sequences, including 2,989 proteins with identified functions. Eleven rRNA operons and 67 tRNAs were also annotated. Plasmids containing a total of 702 coding sequences and having sizes of 130,902 bp, 406,323 bp, 64,131 bp, 5,498 bp, and 4,905 bp were also obtained. The G+C contents of the plasmids range from 36.6% to 37.6%. As expected, several cold shock proteins (Csp) were found in both the chromosome and the second plasmids, explaining the cold adaptability of the strain. Diverse mono- or dioxygenases, dehydrogenases, and cytochromes involved in the hydrocarbon biodegradation pathways were found in the genome, explaining its ability to degrade hydrocarbons. Several lipoprotein, lipopolysaccharide, and polysaccharide biosynthesis genes are present in the genome and are associated with its ability to produce bioemulsifiers.

This is the ninth draft genome for the genus *Exiguobacterium* but the only one isolated from petroleum hydrocarbon-contaminated marine sediment samples. This genome sequence will provide a reference for many further phylogenetic, comparative genomic, metagenomic, and functional studies of this extremophilic genus.

### Accession number(s).

This whole-genome shotgun project has been deposited at DDBJ/EMBL/GenBank under the GenBank accession no. CP022236 to CP022241.
